# Amplified Spontaneous Emission Threshold Reduction and Operational Stability Improvement in CsPbBr_3_ Nanocrystals Films by Hydrophobic Functionalization of the Substrate

**DOI:** 10.1038/s41598-019-54412-7

**Published:** 2019-11-29

**Authors:** Maria Luisa De Giorgi, Franziska Krieg, Maksym V. Kovalenko, Marco Anni

**Affiliations:** 1 Dipartimento di Matematica e Fisica “Ennio De Giorgi”, Università del Salento, Via per Arnesano, 73100 Lecce, Italy; 2 Institute of Inorganic Chemistry, Department of Chemistry and Applied Biosciences, ETH Zürich, CH-8093 Zürich, Switzerland; 3 Laboratory for Thin Films and Photovoltaics, Empa - Swiss Federal Laboratories for Materials Science and Technology, CH-8600 Dübendorf, Switzerland

**Keywords:** Nanoscience and technology, Optics and photonics, Physics

## Abstract

The use of lead halide perovskites in optoelectronic and photonic devices is mainly limited by insufficient long-term stability of these materials. This issue is receiving growing attention, mainly owing to the operational stability improvement of lead halide perosvkites solar cells. On the contrary, fewer efforts are devoted to the stability improvement of light amplification and lasing. In this report we demonstrate that a simple hydrophobic functionalization of the substrates with hexamethyldisilazane (HMDS) allows to strongly improve the Amplified Spontaneous Emission (ASE) properties of drop cast CsPbBr_3_ nanocrystal (NC) thin films. In particular we observe an ASE threshold decrease down to 45% of the value without treatment, an optical gain increase of up to 1.5 times and an ASE operational stability increase of up to 14 times. These results are ascribed to a closer NC packing in the films on HMDS treated substrate, allowing an improved energy transfer towards the larger NCs within the NC ensemble, and to the reduction of the film interaction with moisture. Our results propose hydrophobic functionalization of the substrates as an easy approach to lower the ASE and lasing thresholds, while simultaneously increasing the active material stability.

## Introduction

Lead halide perovskites are receiving an enormous interest in the last few years, mainly driven by their extremely promising photovoltaic properties, ascribed to a combination of large absorption coefficient, low defect density and high charge carrier mobility^1–3^, that recently allowed the demonstration of a certified power conversion efficiency of 25.2%^4^.

Beyond the photovoltaic properties the direct bandgap of lead halide perovskites also allows to obtain high photoluminescence efficiency that stimulated the research in light emitting devices, like light emitting diodes^5–8^.

Moreover optical gain has been demonstrated both in bulk polycrystalline thin films^9–12^ and in perovskite NC thin films^13–19^ both under single and multiphoton absorption^20–22^ and laser demonstrators have been reported with different cavity geometries, including dielectric-metal vertical microcavity^23^, distributed feedback lasers (DFB)^11,24,25^ and whispering gallery mode microlasers^26–30^ and even butterfly wings microstructures^31^.

Despite these promising results and the fast improvements of the performances of the various device demonstrators, the perspectives for applications of lead halide perovskites in diverse optoelectronic devices are currently limited by the poor chemical and morphological stability of these materials. In particular it has been demonstrated that hybrid organic-inorganic perovskites films show a fast degradation due to many factors like vacuum^32^, light exposure^33–37^, temperature^33^ and interaction with oxygen and moisture^38–41^.

In order to improve the operational stability of solar cells based on bulk polycrystalline perovskites films the deposition of a protecting layer between the hole transport layer and the active layer^42–44^ and the employment of a water-resistant electron transport layer^45^, have been proposed.

A further possible strategy to solve these problems is compositional engineering. In this context, fully inorganic perovskites, in which Cesium replaces the organic cations, like methylammonium (MA) or formamidinium (FA), have demonstrated improved thermal stability^46^, efficient electroluminescence^47^ and optical gain^12–14,48,49^.

Moreover, after their first demonstration by L. Protesescu *et al*.^50^, CsPbX_3_ NCs received large attention, thanks to the possibility to widely tune their emission color by acting on their chemical composition and eventually on the quantum size effect. Moreover efficient electroluminescence^51^ and high and tunable optical gain at room temperature have been demonstrated^13^. The peculiar properties of NCs also allowed to improve the photoluminescence intensity stability by acting on the synthesis process^52–54^, by ligands engineering^55–57^ and by incorporation into hydrophobic polymer matrices^58,59^ or microcapsules^60^.

To date, the efforts to enhance the ASE operational stability have been only addressed to organic-inorganic perovskites. In particular the deposition of encapsulating layers, like a PMMA layer, followed by epoxy resin and glass encapsulation in glove box^10^ or a commercial fluoropolymer layer (CYTOP)^11^, has been proposed in MAPbBr_3_ bulk polycrystalline thin films excited by nanosecond pump pulses. Concerning NC films an ASE stability improvement has been recently reported in films of MAPbBr_3_ NCs treated with benzyl alcohol^17^ under femtosecond excitation.

Up to now, no experiments have been thus performed in order to investigate the ASE stability under nanosecond excitation of perovskite NC films and the possible strategies to improve it. This lack of knowledge is particularly relevant for the possible applicative perspectives of perovskites lasers. Even if femtosecond pumping typically allows higher photostability of the active materials, it can be obtained by complex and expensive lasers, not compatible with the development of low costs optically pumped lasers^61^. On the contrary nanosecond pulses with high enough energy to obtain ASE and lasing can be obtained by low cost sources like pulsed laser diodes^62^ and pulsed LEDs^63^. Thus, the understanding and the optimization of ASE and lasing under nanosecond excitation become fundamental for the technological applications, as it mimics the possible working conditions of optically pumped perovskite lasers exploiting cheap and compact pumping sources.

In this report we investigate in details the ASE and gain properties under nanosecond excitation of drop cast CsPbBr_3_ NC thin films and we demonstrate that a clear improvement can be easily obtained by a HMDS hydrophobic functionalization of the glass substrates. In particular, starting from values comparable with the actual state of the art, we observe an ASE threshold decrease down to 45% of the value of the untreated sample, an optical gain increase up to 1.5 times and an ASE operational lifetime increase up to 14 times. These results are ascribed to a closer NC packing in the films on HMDS treated glass, allowing an improved energy transfer toward the larger NCs in the size distribution, and to the reduction of the film interaction with moisture. Our results are expected to be of general validity and provide an easy way to lower the ASE and lasing thresholds, while simultaneously increasing the active material stability.

## Results

### PL, absorbance and PLQY

Our experiment has been performed on CsPbBr_3_ NC with two different sizes, namely (9 ± 2) nm (named NC1 in the following) and (13 ± 2) nm (named NC2) (see Fig. S1). The NCs have been synthesized by adapting the procedure of ref. ^56^, and the films were deposited by drop casting on properly cleaned glass substrates (1 × 1 cm) as described in details in the Supporting Information.

**Figure 1:**
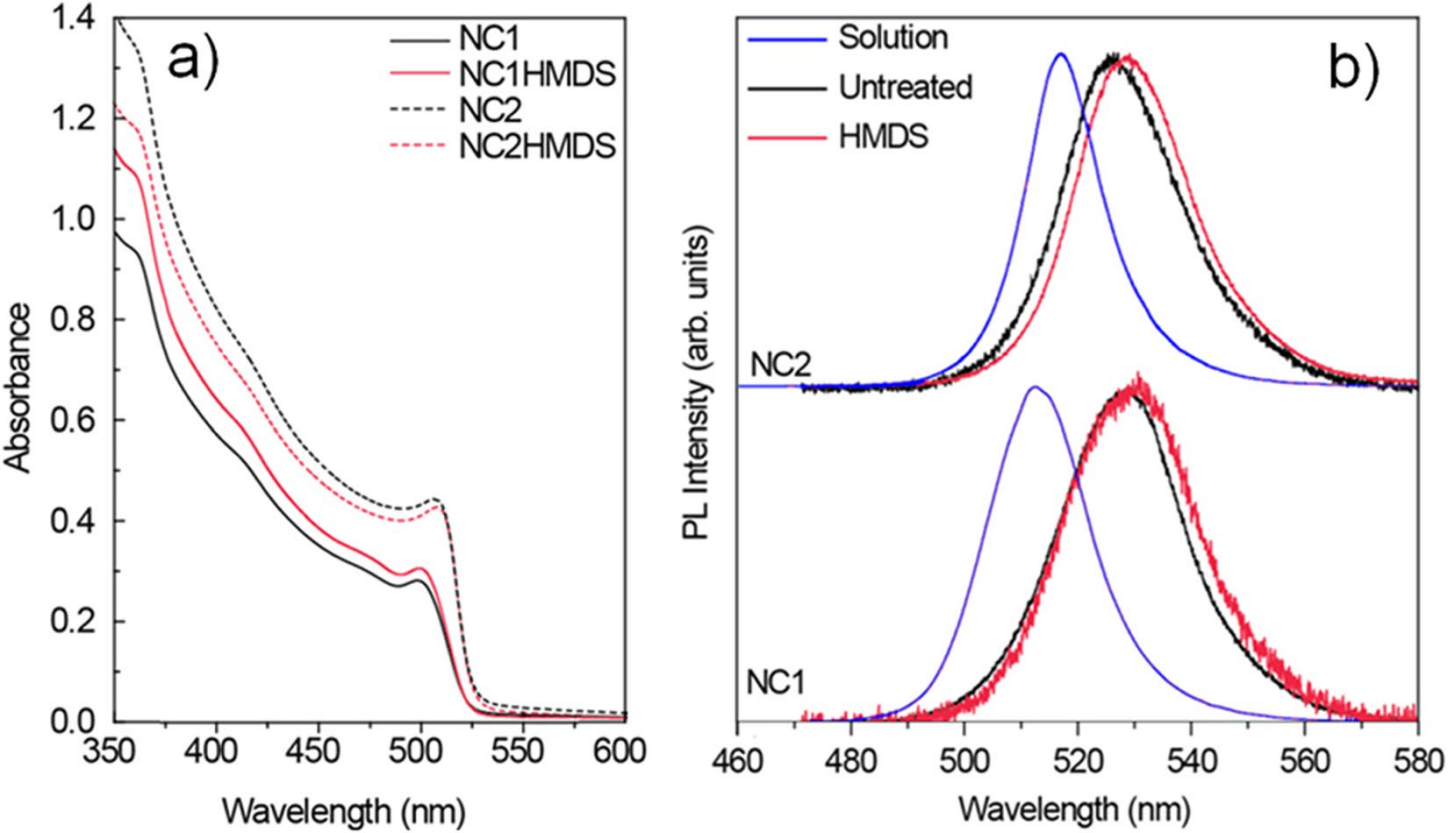
(**a**) Absorbance spectra of NC1 and NC2 films, evidencing a negligible variation due to HMDS functionalization of the substrate. (**b**) Photoluminescence spectra of NC1 and NC2 films deposited on untreated substrates and on HMDS fuctionalized substrates. The PL spectra of the corresponding solution are also reported for comparison.

As a first step in the characterization of the two types of NCs we measured their absorbance, PL spectrum and PLQY in toluene solution. The NC1 solution shows (see Fig. S2) an absorbance spectrum with a clear excitonic peak at 500 nm, a PL spectrum with a peak at 513 nm and a PLQY of about 55%. The NC2 solution shows instead a red-shifted absorbance, with the excitonic peak at 508 nm, a PL peak red-shifted to 517 nm, and a remarkable PLQY of 86%. The absorbance spectra of the NC films (see Fig. 1a) are identical to the ones in solution, and no variations of the lineshape and of the absorbance absolute values are observed between the films deposited on clean glass (named NC1 and NC2 in the following) and the corresponding films deposited on HMDS functionalized substrates (named NC1HMDS and NC2HMDS in the following), evidencing comparable excited states density and comparable thicknesses. The thickness has been also measured from SEM cross sections obtaining a comparable thickness of about 350 nm for samples NC1 and NC1HMDS and of about 425 nm for NC2 and NC2HMDS.

Concerning the PL spectra a clear red-shift of the NC1 and NC2 film spectra with respect to the corresponding solution ones (see Fig. 1b) is observed, and a further red-shift is observed in samples NC1HMDS and NC2HMDS. Concerning the PLQY a decrease of about 2 times is observed in films of NC1 and NC2 with respect to the values in solution, while no systematic differences are observed in the NC1HMDS and NC2HMDS films. These results are summarized in Table 1.

**Table 1:** Luminescence peak wavelength, exciton absorption peak wavelength, and PLQY of the investigated nanocrystals in solution and in films.

Sample	Luminescence peak (nm)	Exciton absorption peak (nm)	Quantum Yield%
NC1 solution	513	500	55
NC2 solution	517	508	86
NC1	529	500	27
NC1HMDS	530	500	35
NC2	526	508	50
NC1HMDS	529	508	51

### Amplified spontaneous emission

**Figure 2:**
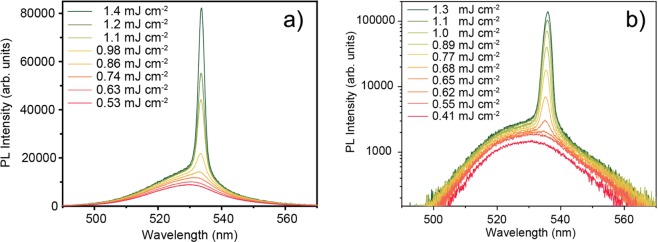
(**a**) Excitation density dependence of the PL spectra of NC1 sample under 3 ns laser pumping. The appearance of the ASE band above about 0.75 mJcm^−2^ is evident. (**b**) Excitation density dependence of the PL spectra of NC1HMDS sample under 3 ns laser pumping. The appearance of the ASE band above about 0.6 mJcm^−2^ is evident.

**Figure 3:**
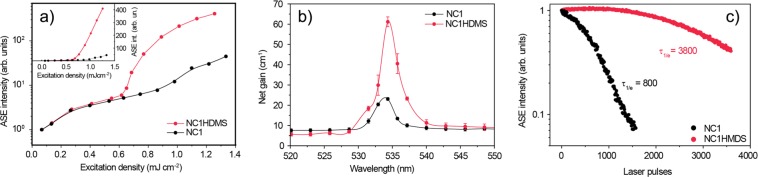
(**a**) Excitation density dependence of the ASE peak intensity of NC1 (black dots) and of NC1HMDS (blue dots) samples. The data have been normalized to 1 at a common excitation density value below both the ASE thresholds (0.075 mJcm^−2^). The lines are guides for the eyes. Inset: The same data plotted with a linear intensity scale, evidencing the strongly different slope of the ASE increase in the two samples. (**b**):Net gain spectrum of NC1 sample at an excitation density of 1.5 mJcm^−2^ (black dots) and of NC1HMDS sample at an excitation density of 2.1 mJcm^−2^ (blue dots). The lines are guide to the eyes. (**c**) ASE peak intensity decrease during continuous laser pumping at an excitation density of 2 times the ASE threshold one in NC1 sample (black dots) and in NC1HMDS (blue dots), evidencing a clear ASE stability improvement in NC1HMDS.

In order to investigate the light amplification properties of the samples we acquired the PL spectra as a function of the excitation density.The PL spectra of sample NC1 as a function of the excitation density (see Fig. 2a) show, at low excitation density, only a spontaneous emission band. By increasing the excitation density, a clear narrow band, with a peak wavelength of about 533.6 nm, appears from 0.75 mJcm^−2^, and progressively dominates.Moreover we observed that the PL intensity at the ASE peak wavelength (see Fig. 3a) grows linearly with the excitation density below 0.75 mJcm^−2^, while at higher densities a superlinear intensity dependence is evidenced by the slope increase. These features are the typical signature of ASE^13,14,64,65^. An ASE threshold of 0.75 mJcm^−2^ has been estimated by determining the average value of the minimum excitation density that allows the observation of the ASE band, measured in 5 different positions on the sample.

It is worthwhile to underline that the ASE performance of NC1 sample is consistent with the current state of the art of CsPbBr_3_ NC films. Indeed, the NC1 ASE threshold value is only about 1. 6 times higher than the best value reported in literature for CsPbBr_3_ under nanosecond pumping^13^, but about 3.4 times lower than the value recently reported under the same excitation condition used in this work^14^.

The excitation density dependence of the PL spectra of the NC1HMDS sample is qualitatively similar (see Fig. 2b), with the presence of the spontaneous emission band for excitation density below 0.6 mJcm^−2^ and the appearance of an ASE band peaked at 536.0 nm at higher excitation densities. Also in this case the appearance of the ASE band results in a clear slope increase of the PL intensity versus the excitation density (see Fig. 3a). The average ASE threshold is 0.68 mJcm^−2^, thus about 11% lower than in the non-functionalized sample NC1.

A second remarkable effect of the HMDS functionalization of the substrate is observed when comparing the ASE intensity dependence on the excitation density (see Fig. 3a). Indeed the ASE intensity increase with the excitation density, above the threshold, is much stronger in the NC1HMDS sample than in NC1. In particular the excitation density increase from 0.075 mJcm^−2^ to 1.25 mJcm^−2^ leads to an intensity increase at the ASE peak wavelength of about 420 times in NC1HMDS, while an increase of only 34 times is observed in NC1.

Concerning the larger NCs, the PL spectra of the NC2 film as a function of the excitation density show a spontaneous emission band (see Fig. S3a) peaked at 526.5 nm, at low excitation density, and the appearance of the ASE band, peaked 536.2 nm, with a threshold of 3.2 mJcm^−2^.

The spectra of NC2HMDS, instead, show a spontaneous emission band redshifted to 530.0 nm at low excitation density and an ASE band peaked at 537.4 nm with a threshold reduced to 1.4 mJcm^−2^ (see Fig. S3b).

The films of larger NCs thus show higher ASE thresholds with respect to the corresponding films with smaller NCs, but the HMDS functionalized substrates lead to a larger ASE threshold reduction (2.3 times).

Moreover, also in these films a strong difference in the ASE intensity increase is observed above the threshold (see Fig. S4a), evidenced by a clearly higher slope in sample NC2HMDS than in NC2.

### Optical gain

In order to further investigate the effects of the HMDS functionalization of the substrate on the light amplification properties of the samples we determined the net gain spectra, starting from the PL spectra dependence of the pumped stripe length at a fixed excitation density above the ASE threshold (variable stripe length method^66^). As in laser applications the active material is typically pumped at an excitation density without compromising the stability, for each sample we preliminarily determined the maximum allowable excitation density to avoid ASE degradation during the measurements. The NC1 sample net gain spectrum has been determined at an excitation density of 1.5 mJcm^−2^ (2 times above the ASE threshold) (see Fig. 3b) and a maximum gain value of 23.1 ± 0.8 cm^−1^ has been obtained at about 534 nm.

A higher stability has been observed for the corresponding functionalized sample, that allowed to determine the gain spectrum at an excitation density of 2.1 mJcm^−2^ (about 3 times the ASE threshold) (see Fig. 3b). A maximum gain value of 61 ± 2 cm^−1^ is obtained at 534.3 nm, thus about 2.7 times higher than the NC1 maximum gain at an excitation density only 1.4 times higher. In order to compare the gain values with the literature we observe that gain values up to 12.9 cm^−1^ at 6.0 mJcm^−2^ have been recently found in CsPbBr_3_ NC drop cast films in the same excitation conditions used in the current experiment^14^, evidencing that the NC1HMDS has a 5 times larger gain at an excitation density 3 times lower.

With the aim of comparing the intrinsic gain properties of the two samples we estimated the net gain value of NC1HMDS at an excitation density of 1.5 mJcm^−2^ (at which the gain of NC1 has been measured).

Remembering that the net gain linearly increases with the excitation density, up to gain saturation, and that the net gain is 0 at the ASE threshold excitation density (0.68 mJcm^−2^ for NC1HMDS sample), we evaluated a lower limit for the net gain of NC1HMDS. It results that the gain of NC1HMDS at an excitation density of 1.5 mJcm^−2^ cannot be lower than 35 cm^−1^, that is about 1.5 times higher than the measured net gain of the NC1 sample.

Concerning the larger NCs samples the high ASE threshold value of the NC2 sample prevented the measurement of the net gain spectrum, due to a fast ASE degradation also at 6.4 mJcm^−2^ (thus about 2 times above the ASE threshold). Sample NC2HMDS showed instead a maximum gain value of about 56 cm^−1^ at an excitation density of 2.8 mJcm^−2^ (2 times above the ASE threshold), thus roughly comparable with the one of NC1HMDS, but at excitation density about 1.4 times larger.

### ASE operational stability

The ASE operational stability has been investigated by measuring the PL spectra above the ASE threshold during continuous pumping in air. Considering that in real laser applications a device is operated at an excitation density that allows good light amplification, and remembering that the ASE threshold quantify the minimum excitation density necessary to have light amplification, we measured the ASE intensity decrease during continuous laser pumping at an excitation 2 times larger than the ASE threshold for all the samples. In particular, the excitation density was set at 1.5 mJcm^−2^ and 1.4 mJcm^−2^ for samples NC1 and NC1HMDS, respectively and at 6.4 mJcm^−2^ and 2.8 mJcm^−2^ for samples NC2 and NC2HMDS, respectively.

For sample NC1 we observe (see Fig. 3c) a continuous decrease of the ASE intensity during pumping. The ASE operational lifetime, quantified as the number of pump laser pulses necessary to have an ASE intensity reduction of a factor e (1/e lifetime) is about 800 pulses. A very evident stability improvement is observed in sample NC1HMDS (see Fig. 3c), showing a stable ASE intensity up to about 1000 laser pulses, and then a progressive intensity decrease at longer times. The 1/e lifetime has been quantified in about 3800 pulses from an extrapolation of the experimental data, thus about 5 times higher than the one of NC1. In particular after 1500 pulses the ASE intensity of the NC1HMDS sample is still 97% of the initial value, while it is only 7% of the initial value in sample NC1.

Sample NC2 shows (see Fig. S4b) a quick ASE intensity decrease with a 1/e lifetime of only 140 pulses, followed by a slower intensity decrease starting after about 1000 pulses. In this regime the ASE band is no longer present in the spectra, thus the slower degradation is due to the spontaneous emission intensity decrease, with a lifetime of 17600 pulses. Sample NC2HMDS shows an evidently higher ASE intensity stability, with a 1/e lifetime of 1950 pulses (thus 14 times longer than NC2), followed by ASE disappearance after about 5000 pulses and a slower spontaneous emission relaxation with a lifetime of about 27000 pulses. For the sake of comparison we observe that the only investigation on the ASE operational stability in CsPbBr_3_ NC films has been performed under femtosecond pumping^67^, evidencing a ASE 1/e lifetime of about 5 × 10^5^ pulses. A reasonable estimate of the contribution of the use of nanosecond pumping instead that ultrafast pumping can be done by comparing the results in literature on bulk polycrystalline thin films of organic-inorganic perovskites. In particular the complete suppression of ASE was observed in MAPbBr_3_ films after about 9000 nanosecond (1 ns) pulses at 320 *μ*Jcm^−2^, even if the films were encapsulated by ultraviolet curing adhesive, covered by glass in the nitrogen-filled glovebox and pumped only 1.05 times above the threshold^10^. On the contrary stable ASE intensity for at least 9 × 10^7^ femtosecond pulses was reported in MAPbI_3_ films^9^. Considering that MAPbBr_3_ is known to show better photostability than MAPbI_3_^68^ these results suggest that pumping with nanosecond pulses reduces the ASE operational lifetime of at least 4 order of magnitudes with respect to femtosecond pumping. Assuming a similar effect on CsPbBr_3_ NC films we should thus expect an ASE operational lifetime on the scale of tens of pulses. Thus we can conclude that the evidence of a ASE 1/e lifetime increase to the regime of thousands of nanosecond pulses in non encapsulated films operating in air demonstrates a remarkable photostability of the samples deposited on HMDS functionalized substrates.

### Sample morphology

**Figure 4:**
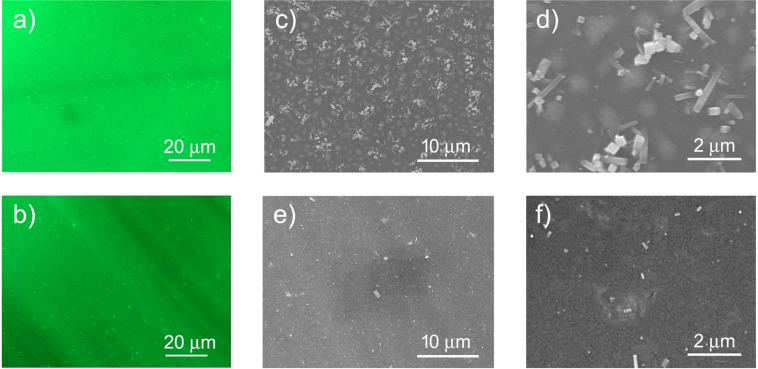
108 × 81 *μ*m^2^ fluorescence map of NC1 sample (**a**) and of NC1HMDS (**b**). SEM images of sample NC1 at a magnification of 3000 times (**c**) and 13000 times (**d**) and of sample NC1HMDS at a magnification of 3000 times (**e**) and 13000 times (**f**).

We also investigated the local photoluminescence properties of the films by fluorescence microscopy mapping and the film morphology by SEM microscopy. The fluorescence map of sample NC1 (see Fig. 4a) evidences an almost uniform emission intensity, with small bright clusters with a typical size below 1 *μ*m. The map of NC1HMDS (Fig. 4b) instead shows evident stripes with different PL intensity likely related to thickness fluctuations, and a reduced density of bright clusters.

The SEM image of the NC1 sample at a magnification of 3000×(see Fig. 4c) allows to clearly observe a uniform film with a dense distribution of clusters on the surface, corresponding to the bright clusters visible in the fluorescence maps, that at higher magnification (see Fig. 4d) evidence a regular parallelepiped shape, with a typical short edge length of about 300 nm and a long edge length below 2 *μ*m. The regular shape of these clusters suggests that these might be microcrystallites of CsPbBr_3_ formed by the aggregation and subsequent fusion of NCs^69^.

Moreover a clear clustering of the NCs is also observed in the background evidenced by the presence of rounded brighter regions with a characteristic size of about 1 *μ*m. The surface roughness, obtained from AFM images of a 10 *μ*m × 10 *μ*m region of the sample is about 140 nm.

The SEM image of the NC1HMDS shows a clear reduction of the density of rectangular clusters on the film surface and a considerable decrease of their size. Higher uniformity is also observed in the film in which no evidence of NC clustering is present (see Fig. 4e,f), and the surface roughness is decreased to 49.7 nm.

The fluorescence map of the NC2 films (see Fig. S5a) evidences an intensity modulation of the background fluorescence, likely related to thickness fluctuations, and bright clusters on the few microns scale. The NC2HMDS sample (see Fig. S5b) still shows the presence of brighter and darker stripes, related to thickness fluctuations, with the further presence of darker elongated regions with a lateral size of about 1 micron, and a length of few tens of microns. Moreover, a reduction of the micrometer-sized cluster density is observed.

The SEM image of the NC2 sample (see Fig. S5c) at a magnification of 3000X allows to observe a slightly rough film surface with rounded clusters on the few micron scale, while at higher magnification (see Fig. S5d) a grainy structure suggests the presence of nanocrystals clustering on the submicrometric scale. The surface roughness is 63.1 nm.

The NC2HMDS SEM image at a magnification of 3000×(see Fig. S5e) again allows to observe a reduced aggregation on the micron scale and a higher overall uniformity of the film. The SEM image at higher magnification (see Fig. S5f) shows a lower contrast with respect to the NC2 one, evidencing a higher film uniformity. The surface roughness is 58.3 nm.

## Discussion

In order to rationalize the origin of the observed improvement of ASE threshold, intensity and stability, due to the HMDS functionalization of the substrate, we start by observing that the final ASE properties can be affected by several extrinsic factors, like waveguide thickness^70,71^ and active film uniformity^72,73^, beyond the intrinsic gain cross section of the active layer.

Nevertheless we can exclude waveguiding differences along the films, as NC1 and NC1HMDS have comparable thickness, as well as NC2 and NC2HMDS.

We also exclude any relevant role of the film micromorphology as, while the lower microcrystal density of NC1HMDS with respect to NC1 could lead to the guess of lower waveguide losses improving the ASE^72^, samples NC2 and NC2HMDS have comparable microcrystals density but clearly different ASE properties.

A further explanation could be a general improvement of the emission properties of the NCs due to passivation of surface defects. However also this attribution can be excluded by observing that NC1HMDS sample shows higher PLQY than the NC1 and better ASE properties but NC2 and NC2HMDS have basically identical PLQY, but show large differences in the ASE properties.

The microscopic origin of the observed effects of the HMDS substrate functionalization can be understood by remembering that the ASE properties are determined by the net gain *g*′ values of the waveguide, given by *g*′ = *g* − *α* = *σN* − *α*, where *g* and *α* are the gain and losses coefficients, *σ* the gain cross section and *N* the volumetric density of population inversion, respectively. Assuming a uniform gain value across the pumped stripe and almost steady state excitation (as the laser pump pulse length, Δ*t*, is longer than the typical ASE lifetime *τ*_*ASE*_^9,13^), the collected intensity can be written as:

1 $$I(\lambda )=\frac{{I}_{0}}{g^{\prime} }({e}^{g^{\prime} L}-1)$$

where *L* is the pump stripe length. Moreover, for a given absorbed excitation density *D*, N can be written as:

2 $$N={\eta }_{pump}\frac{D}{dh\nu }\frac{{\tau }_{ASE}}{\Delta t}=A\cdot D$$

3 $$A=\frac{{\eta }_{pump}}{dh\nu }\frac{{\tau }_{ASE}}{\Delta t}$$

where *d* is the film thickness, *hv* the pump photon energy and *η*_*pump*_ is the pumping efficiency (i.e. the fraction of absorbed photons that generate an exciton in the emitting state). From the above equations it follows that the ASE threshold is given by the excitation density that allows to have *g*′ = 0, thus *g* = *α*, and it is thus determined by an interplay between the intrinsic gain properties of the material and the waveguide losses. On the contrary the excitation density dependence of the ASE intensity is determined by the excitation density dependence of *g*, and thus by the *A* value.

Imposing the threshold conditions at the experimental ASE threshold values and comparing the experimental ASE intensity increase with the one predicted from Eq. 1 (see Fig. S6), we obtained an estimate of the A and *α* values (see Table 2) in all the samples.

**Table 2:** Estimate of the A and *α* values for the investigated samples.

Sample	A (cm^−1^mJ^−1^)	*α* (cm^−1^)
NC1	16.5	12.4
NC1HMDS	26.2	17.8
NC2	3.7	11.8
NC2HMDS	10.0	14.0

The obtained values suggest that the HMDS functionalization of the substrate leads to a slight increase of the waveguide losses (about 1.4 times in the smaller NCs and 1.2 times in the larger ones) and to a strong increase of the gain at any given excitation density (about 1.6 times for the smaller NCs and about 2.7 times for the larger ones), which is responsible for the observed stronger increase of the ASE intensity as a function of the excitation density. Considering the simplicity of the used model the estimated increase of gain is in excellent agreement with the one observed between samples NC1 and NC1HMDS.

The origin of the losses increase can be understood thanks to the confocal PL maps that show more evident thickness irregularities on the tens of microns scale in the NC1HMDS and NC2HMDS samples with respect to NC1 and NC2, leading to higher scattering induced waveguide propagation losses.

Concerning the origin of the increased gain we observe that the gain value at a given absorbed excitation density is determined by the *A* value, thus three effects can determine a gain increase, namely a thickness decrease, an ASE lifetime increase and an excitation efficiency increase.

As the samples have similar thickness we can exclude any role of the sample thickness. Analogously, comparable PLQY values imply similar balance between radiative and non radiative recombination channels, suggesting similar ASE lifetime in the two pairs of samples. This analysis thus suggests that the differences between the samples deposited on HMDS functionalized substrates with respect to the ones deposited on untreated substrates is related to the excitation efficiency of the finally emitting excited state. This conclusion is supported by the observed PL red-shift between the solutions and the NC1 and NC2 samples, evidencing that energy migration within the inhomogeneously broadened excited state density takes place between the pump photons absorption and the PL emission, as already observed and discussed in similar systems^74^. The further red-shift in the NC1HMDS and NC2HMDS with respect to the corresponding non treated films clearly implies an increased energy migration efficiency towards the larger NCs in the size distribution, emitting in the high wavelength tail of the ensemble PL spectrum. This effect actually increases the exciton generation efficiency in the larger NCs, due to the contribution of exciton migration from the smaller ones, resulting in a larger population inversion density at any given absorbed pump density. This conclusion is also supported by the SEM images: the absence of NC aggregation in the active film, and thus a more uniform NC mixing, in NC1HMDS and NC2HMDS samples, allows an improved exciton migration in the film.

We also observe that more uniform packing of NCs in films deposited on HMDS functionalized substrates has been also reported in colloidal II-VI NCs films^75^. The origin of this effect can be ascribed to the hydrophobicity of the HMDS treated substrates, evidence by the water droplets contact angle increase (see Supporting Information). Also the NCs are hydrophobic because their surface is covered with long-alkyl-chain ligands surface^76^, and toluene is a hydrophobic solvent. Thus the hydrophobic functionalization of the substrate allows an improvement of the substrate wetting from the NCs solution during the film formation, allowing a more uniform NCs arrangement in the film.

Concerning the origin of the observed strong increase of the ASE operational stability we observe that it is widely accepted that the main CsPbBr_3_ degradation process is related to the interaction with moisture^69,76,77^, and that several results on the photostability improvements due to the coating with hydrophobic layers, blending in hydrophobic polymer^77^ or even HMDS addition to the growth solutions^57^. In our experiment the HMDS is only present on the substrate surface, thus we can exclude any surface passivation of the NCs far from the substrate surface and the formation of a direct barrier against the moisture diffusion in the film. Anyway it has been demonstrated that a hydrophobic polystyrene layer causes a clear increase of the hydrophobicity of a 200 nm spiro-OMeTAD layer deposited on it^43^, clearly evidencing that a hydrophobic layer can modify the wettability also far from its surface. We thus ascribe the improved ASE operational lifetime to a hygrophobic behavior of the films, induced by the hydrophobic nature of teh functionalized substrates. We also observe that a lower reactivity of the NC1HMDS and NC2HMDS films with moisture is strongly suggested by the clearly reduced size and density of the microcrystals, whose formation is ascribed to moisture induced dissolution and recrystallization^69^.

In conclusion we demonstrated that a hydrophobic functionalization of the substrate allows a strong improvement of the ASE properties of CsPbBr_3_ NC films, evidenced by a ASE threshold decrease, an increase of the ASE intensity dependence on the excitation density, and a considerable increase of the ASE operational lifetime. These effects are ascribed to a closer packing and a reduced aggregation of the NCs in the film deposited on HMDS functionalized substrates, enhancing the exciton transfer from small to large NCs in the film, and to a reduced interaction with moisture. Our results demonstrate that the ASE and gain properties of perovskites Ncs films can be improved without the need of encapsulation or inclusion in hydrophobic matrices.

## Methods

The NCs have been synthesized by adapting the procedure of Ref. ncfran, as described in details in the Supporting Information.

Optical absorption UV-Vis absorption spectra were collected by using a Jasco V670 spectrophotometer in transmission mode.

A Fluorolog iHR 320 Horiba Jobin Yvon spectrofluorometer equipped with a PMT detector was used to acquire steady-state photoluminescence (PL) spectra from solutions.

Absolute PL Quantum Yield (QY) of films and solutions were measured by a Quantaurus-QY Absolute PLQY spectrometer from Hamamatsu.

Transmission Electron Microscope (TEM) images were collected by using Hitachi HT7700 TEM operated at 100 kV and processed by using Fiji^78^.

Scanning Electron Microscope (SEM) images were collected by a JEOL JSM-6480LV SEM, operated at 20 kV. In order to prevent charging effects the samples have been metalized by depositing a 10 nm thick gold film on the surface by sputtering in Ar atmosphere at a pressure of 10^−1^ mbar, with a Quorumm Technologies- Emitech K550x sputter coater.

Atomic Forme Microscope (AFM) measurements were performed by a Nanosurf- EasyScan DFM AFM.

The local PL properties were investigated by confocal laser spectroscopy using a Nikon Eclipse C1 Confocal Laser Scanning inverted microscope by exciting the samples with the 488 nm line of an Argon laser. The PL was collected in backscattering configuration and detected, in the range 530 ± 20 nm, by a photo-multiplier.

Contact angle measurements have been performed by a DataPhysics OCA 35 goniometer, by dispensing distilled water droplets with volumes of 1−2 *μ*L and averaging over 10 different droplets for each substrate.

In the PL and optical gain measurements on the films we used as excitation source a LTB MNL 100 nitrogen laser, delivering 3 ns pulses at 337 nm, with a maximum peak energy of 155 *μ*J. The pump laser has been focused by a cylindrical lens in order to obtain a rectangular stripe with length up to 4 mm and width of 80 *μ*m. The laser excitation density has been varied by a variable neutral filter. The samples emission was collected, after waveguiding along the pumped stripe, from the sample edge. An Acton 750 spectrometer was used for the spectral dispersion, and an Andor Peltier cooled CCD as detector. The spectral resolution was 0.5 nm. In order to limit photodegradation the PL and gain measurements have been performed with the sample under low vacuum, while the ASE operational stability has been investigated with the films in air atmosphere.

## Supplementary information

Supplementary Information
