# Supplementary material for: Amplified Spontaneous Emission Threshold Reduction and Operational Stability Improvement in CsPbBr3 Nanocrystals Films by Hydrophobic Functionalization of the Substrate
Source: Sci Rep. 2019 Nov 29;9:17964. doi: 10.1038/s41598-019-54412-7 (PMC6884571; doi:10.1038/s41598-019-54412-7)
Supplement: Supplementary file 1 — Supplementary Information [file 41598_2019_54412_MOESM1_ESM.pdf]

Supporting Information

Amplified Spontaneous Emission Threshold Reduction and Operational Stability Improvement in $CsPbBr_3$ Nanocrystal Films by Hydrophobic Functionalization of the Substrate

Maria Luisa De Giorgi[1], Franziska Krieg[2,3], Maksym V. Kovalenko[2,3], and Marco Anni[1*]

[1]*Dipartimento di Matematica e Fisica "Ennio De Giorgi",Università del Salento, Via per Arnesano, 73100 Lecce, Italy.*

[2]*Institute of Inorganic Chemistry, Department of Chemistry and Applied Bioscience, ETH Zürich, CH-8093 Zürich, Switzerland.*

[3]*Laboratory for Thin Films and Photovoltaics, Empa - Swiss Federal Laboratories for Materials Science and Technology, CH-8600 Dübendorf, Switzerland.*

**corresponding author's email: marco.anni@unisalento.it*

Nanocrystals synthesis

The nanocrystals have been synthesized by adapting the procedure described in Ref. [S1].

Chemicals
Lead acetate trihydrate, Oleic acid, Bromine, 1-Octadecene, 3-(N,N-dimethyloctadecylammonio)-propanesulfonate, Toluene and Ethanol were purchased from Sigma Aldrich; Cesium carbonate from Fluorochem; Trioctyl phosphine from STREM; Hexamethyldisilazan (HMDS) from Acros; Acetone, Hexane and Ethylacetate from Fisher.

3-(N,N-dimethyloleylammonio)-propanesulfonate
was prepared according to the 2 step protocol described in Ref. [S1].

Cesium oleate (0.4 M)
1.628 g of Cs2CO3 (10 mmol, 2 eq Cs) and 5 mL of oleic acid (16 mmol, 0.8 eq) were evacuated in a three-neck flask along with 20 mL of ODE at room temperature until the first gas evolution subsides and then further evacuated at 25-120 °C for 1 hour.

Lead (II)-oleate (0.5 M)
4.6066 g of lead (II) acetate trihydrate (12 mmol, 1 eq) and 7.6 mL of oleic acid (24 mmol, 2 eq) were evacuated in a three-neck flask along with 16.4 mL of ODE at room temperature until the first gas evolution subsides and then further evacuated at 25-120 °C for 1 hour.

$TOPBr_2$ (0.5 M)
TOP (6 mL, 13 mmol) and Br2 (0.6 mL, 11.5 mmol) were reacted under inert atmosphere and diluted with toluene (18.7 mL) once the reaction was cooled to room temperature.

$CsPbBr_3$ small nanocrystals (NC1)
PbOleate (5 mL, 2.5 mmol), Cs Oleate (4 mL, 1.6 mmol), 3-(N,N-dimethyloleylammonio)-propanesulfonate (0.215 g, 0.5 mmol) were mixed with 50 mL 1-Octadecene and heated to 120°C under vacuum, where the atmosphere was changed to Argon. The temperature was let to cool to 100°C where $TOPBr_2$ was injected (5 mL, 5 mmol of halides). The reaction was cooled down immediately by an ice bath.

Isolation
To the crude solution (64 mL) 128 mL of ethyl acetate were added and the NC s were precipitated by centrifugation at 29500 xg (g is the earth gravitation constant) for 10 minutes.

Washing
The NCs were dispersed in 20 mL of toluene and precipitated with 40 mL of ethyl acetate and centrifugation at 29500 xg for 1 minute. During the second and third purification step solvents were reduced by factor 2 for each step. After the last precipitation the NCs were dispersed in toluene and centrifuged once more at 29500 xg for 10 min resulting in a 5 mg/mL colloidal solution.

$CsPbBr_3$ large nanocrystals (NC2)
PbOleate (5 mL, 2.5 mmol), Cs Oleate (4 mL, 1.6 mmol), 3-(N,N-dimethyloctadecylammonio)-propanesulfonate (0.215 g, 0.5 mmol) were mixed with 50 mL 1-Octadecene and heated to 120°C under vacuum, where the atmosphere was changed to Argon. The temperature was further elevated to 180°C where $TOPBr_2$ was injected (5 mL, 5 mmol of halides). The reaction was cooled down immediately by an ice bath.

Isolation
The crude solution was centrifuged at 29500 xg for 10 min the precipitate was collected and the supernatant discarded.

Washing
The NCs were dispersed in 20 mL of toluene and precipitated with 40 mL of ethyl acetate and centrifugation at 29500 xg for 1 minute. During the second and third purification step solvents were reduced by factor 2 for each step. After the last precipitation the NCs were dispersed in toluene and centrifuged once more at 29500 xg for 10 min resulting in a 8.6 mg/mL colloidal solution.

Substrates preparation

As a first step the glass substrates were sonicated in soap water, rinsed with deionized water, and dried by a compressed air jet (this procedure was repeated twice). Then they were sonicated in ethanol and dried with air jet then sonicated in acetone and dried with air jet.

The substrates then were either sonicated in hexane and dried with air jet before the films were deposited by drop casting 10 μL of the NC colloidal solution on substrates (films NC1 and NC2), or functionalized by drop casting 30μL of HMDS onto the glass substrate and letting it dry. The adsorbed HMDS was then annealed at 150 °C for 30 minutes and the films cooled back to room temperature during 1 hour before drop casting 10 μL of the same NC colloidal solution on the substrates (films NC1HMDS and NC2HMDS).

Substrates characterization

The wettability of the substrates have been investigated by water contact angle measurements. The equilibrium contact angle, averaged over 10 different distilled water droplets. is 60.7° for the untreated substrate, evidencing a wetting behavior. The HMDS functionalized substrates showed instead an average equilibrium contact angle of 89.0°, and are thus hydrophobic.

Nanocrystals size distribution

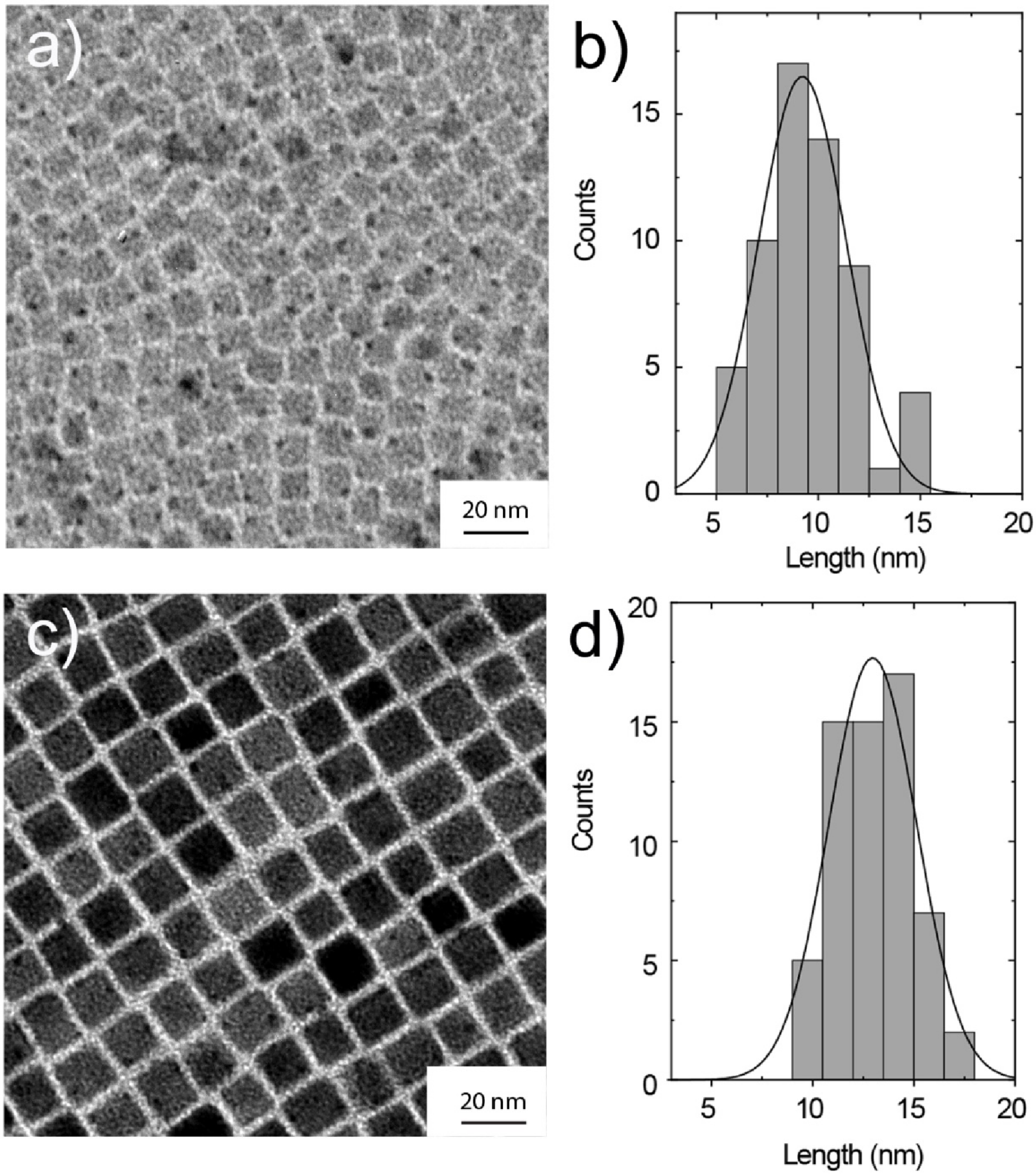

Figure S1: TEM image of the NC1 (a) and NC2 (c) nanocrystals and corresponding histograms of the size distribution (b and d). The continuous line is the best fit curve with a Gaussian distribution.

Absorption and photoluminescence spectra

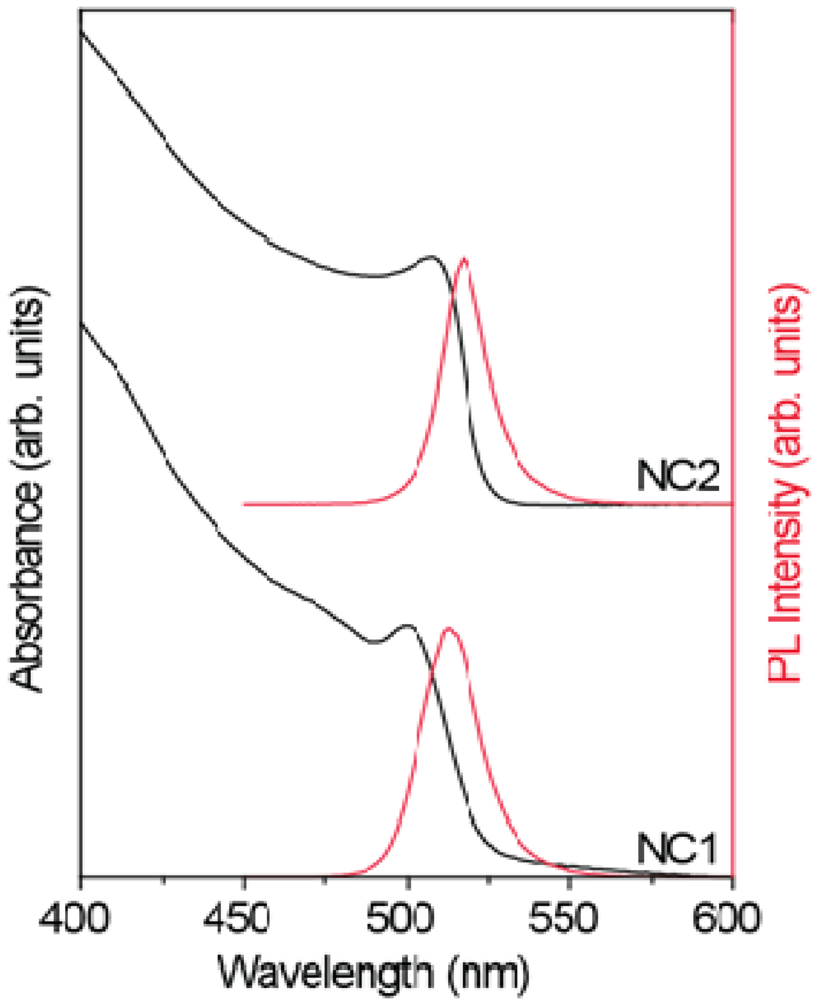

Figure S2: Absorbance and PL spectra of NC1 and NC2 in toluene solution. The spectra are normalized to 1 at the PL peak wavelength and at the exciton absorption peak for clarity.

Amplified Spontaneous Emission properties of the NC2 samples

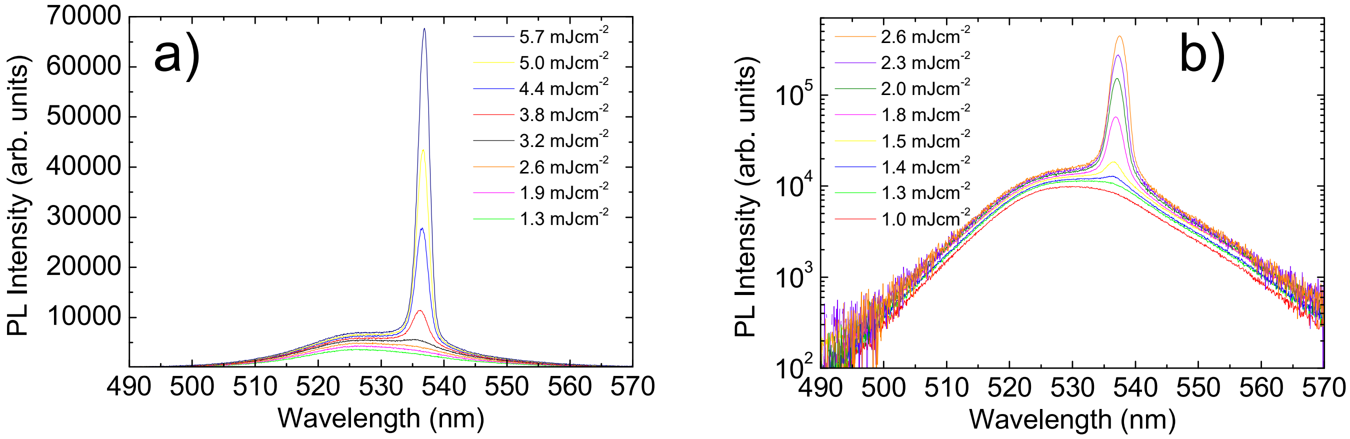

Figure S3. a): Excitation density dependence of the PL spectra of NC2 sample under 3 ns laser pumping. The appearance of the ASE band above 3.2 mJ cm^{-2} is evident. b): **:** Excitation density dependence of the PL spectra of NC2HMDS sample under 3 ns laser pumping. The appearance of the ASE band above about 1.4 mJ cm^{-2} is evident.

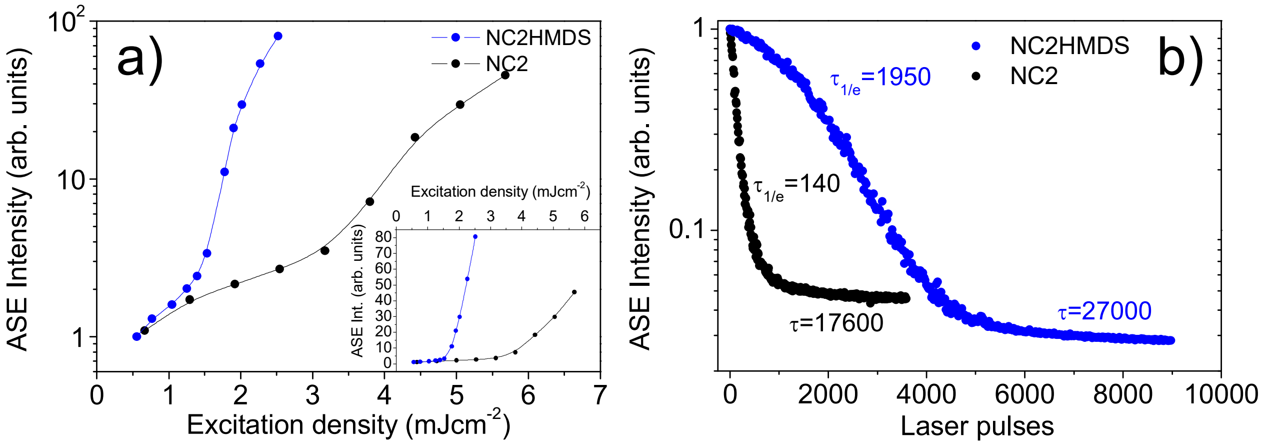

Figure S4. a)**:** Excitation density dependence of the ASE peak intensity of the NC2 (black dots) and of the NC2HMDS (blue dots) samples. The lines are guide for the eyes. Inset: The same data plotted with a linear intensity scale, evidencing the strongly different ASE increase in the two samples. b): ASE peak intensity decrease during continuous laser pumping at an excitation density of 6.4 $mJcm^{-2}$ in the NC2 sample (black dots) and of 2.8 $mJcm^{-2}$ in the NC2HMDS sample (blue dots). The initial fast degradation is due to the ASE progressive quenching with pumping time while at longer times a slower degradation is observe, due to the spontaneous emission quenching.

NC2 samples morphology

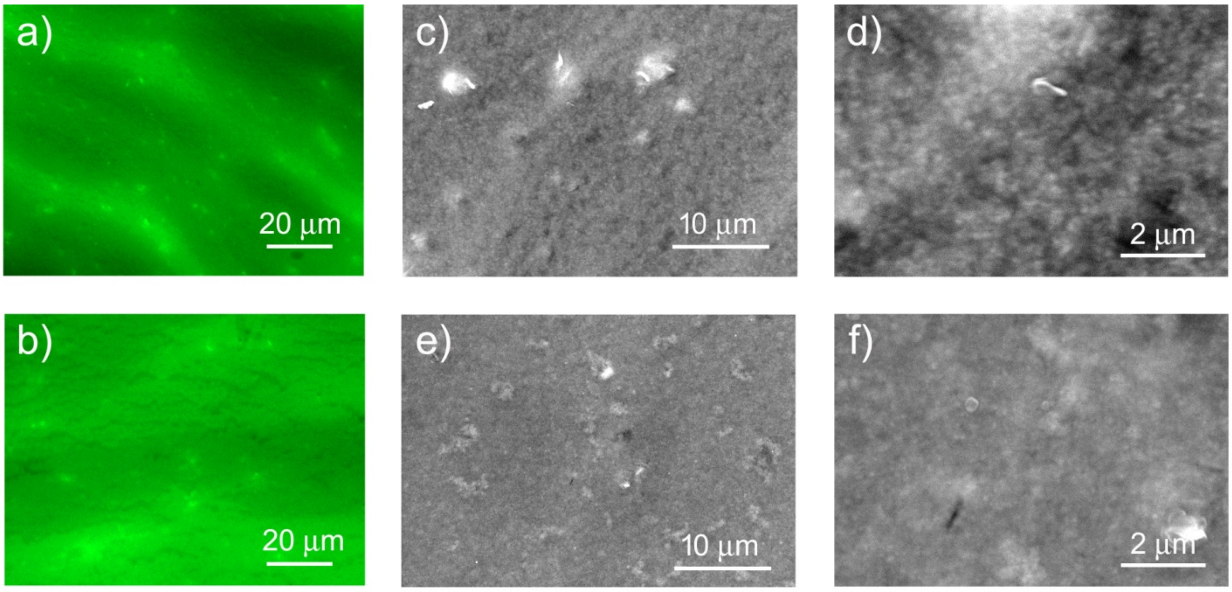

Figure S5: 108*81 μm^2 fluorescence map of the NC2 sample (a) and of the NC2HMDS (b). SEM images of sample NC2 at a magnification of 3000 times (c) and 13000 times (d) and of the sample NC2HMDS at a magnification of 3000 times (e) and 13000 times (f).

Simulated excitation density dependence of the ASE intensity

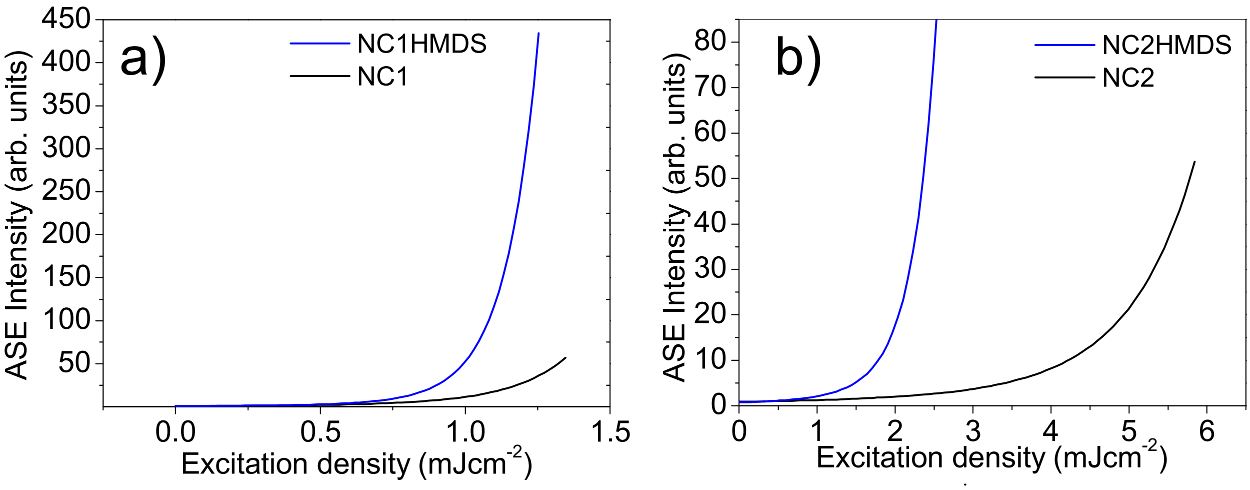

Figure S6: Simulated ASE intensity dependence on the excitation density in samples NC1 and NC1HMDS (a) and NC2 and NC2HMDS (b).

References

[S1] Krieg, F.; Ochsenbein, S. T.; Yakunin, S.; ten Brinck, S.; Aellen, P.; Süess, A.; Clerc, B.; Guggisberg, D.; Nazarenko, O.; Shynkarenko, Y.; Kumar, S.; Shih, C.-J.; Infante, I.; and Kovalenko, M. V.; Colloidal CsPbX3 (X = Cl, Br, I) Nanocrystals 2.0: Zwitterionic Capping Ligands for Improved Durability and Stability, *ACS Energy Letters* **2018** *3*, 641-646.
